# Ectopic thyroid tissue in the iris: a case report

**DOI:** 10.1186/s12886-021-02073-4

**Published:** 2021-08-28

**Authors:** Helena Wagner, Claudia Auw-Hädrich, Martin Werner, Thomas Reinhard

**Affiliations:** 1 Eye Center, Medical Center –University of Freiburg, Faculty of Medicine, University of Freiburg, Killianstraße 5, 79106 Freiburg im Breisgau, Germany; 2 Department of Pathology, Medical Center - University of Freiburg, Faculty of Medicine, Breisacher Str. 115A, 79106 Freiburg im Breisgau, Germany

**Keywords:** Ectopic thyroid tissue, Choristoma, iris tumour, Endothelial decompensation, Keratoplasty, Case report

## Abstract

**Background:**

Ectopic thyroid tissue in the iris, also known as a thyroid glandular epithelial choristoma of the iris, has only been described twice in the literature. In both cases it remained asymptomatic.

**Case presentation:**

A 67-year-old female patient presented for the first time in mid-2017 with corneal endothelial decompensation, with a history of complicated cataract surgery and IStent® implantation. Slit lamp microscopy showed endothelial decompensation, pseudophakia, anterior synechiae and a whitish iris tumour adhering to the endothelium. The latter had existed since childhood. Given these findings, reduced visual acuity of hand movement perception and an intraocular pressure of 23 mmHg, we performed a keratoplasty combined with an en bloc resection of the iris tumour at 9 o’clock and sector iridectomy at the end of 2019. Histological and immunohistological examination of the iris tumour unexpectedly revealed thyroid tissue. After the procedure described above, the patient had an increase in visual acuity while the graft stayed clear and the eye showed no evidence of tumour recurrence or other complications.

**Conclusions:**

We report a third case of ectopic thyroid tissue in the iris. Both previous cases remained asymptomatic, whereas in our case, size and location of the ectopic thyroid tissue contributed to a more complex cataract surgery resulting in endothelial decompensation. Therefore, in such cases appropriate patient information should be provided prior to cataract surgery. Furthermore, careful histological examination and examination of the thyroid is important to exclude malignant diagnoses such as a metastasis of a follicular thyroid carcinoma.

## Background

Ectopic thyroid tissue in the iris, also known as a thyroid glandular epithelial choristoma of the iris, has only been described twice in the literature [1, 2]. In both cases it remained asymptomatic. In our case, size and location of the tissue contributed to a more complex cataract surgery and thus, secondarily, to endothelial decompensation.

## Case presentation

A 67-year-old female patient first presented to our hospital in 2017 with a corneal decompensation following complex cataract surgery and iStent® implantation in her right eye a few months prior. A tumour in the anterior chamber was found to have existed since childhood (or even since birth), which had been monitored ophthalmologically for over 20 years. An iris leiomyoma or a juvenile xanthogranuloma had been suspected. In addition, there was a history of open-angle glaucoma on both eyes. In general, the patient suffered from depression, hearing loss on both sides and hypothyroidism following multiple strumectomies performed for recurrent goitre (two between 1960 and 1980 and one around 2000).

**Fig. 1:**
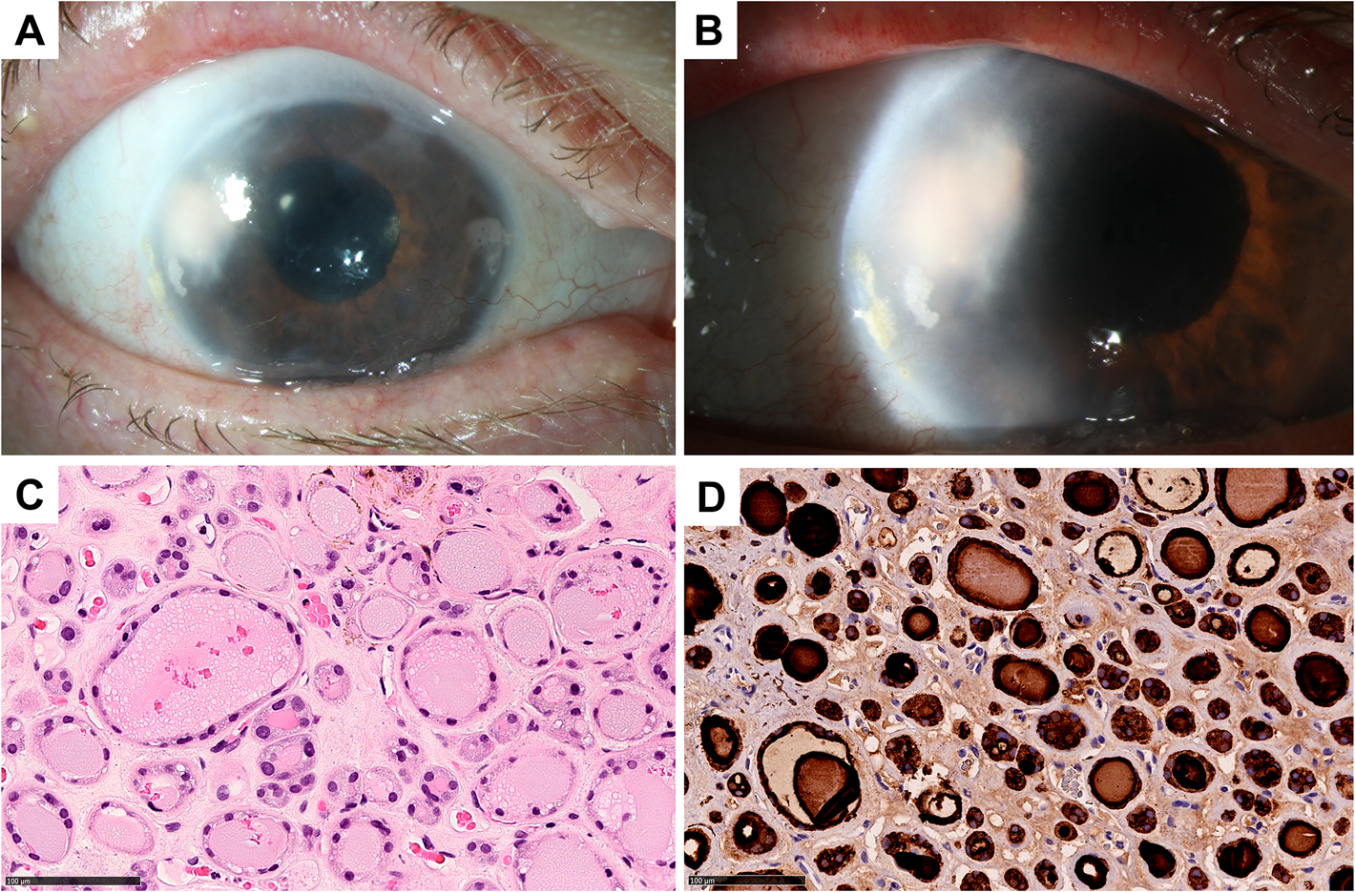
Clinical and histological presentation. **A** and **B** Corneal oedema with an underlying whitish tumour in the anterior chamber at 9–10 o’clock and partial anterior synechiae. **C** The tumour tissue consists of glandular-like formations. Small and large lumina are lined by cubic and by flat epithelial cells. **D** The epithelial cells and the contents of the lumina stained positive with antibodies against thyroglobulin.

At presentation the patient complained of deterioration of vision in her right eye since cataract surgery and iStent® implantation. In the examination, the visual acuity was reduced to the perception of hand motions on the right eye, while it was 1.0 (decimal acuity, equivalent to 20/20) on the left eye. Slit-lamp microscopy revealed not only endothelial decompensation and pseudophakia but also anterior synechia and a whitish iris tumour originating from the iris adhering to the endothelium (Fig. 1 A and B).

The anterior synechia formed by the tumour with the endothelium excluded a Descemet’s membrane endothelial keratoplasty as a therapeutic option. We therefore decided to perform a penetrating keratoplasty with en-bloc resection of the iris tumour and sector iridectomy at the end of 2019.

The tissue obtained was histologically examined after surgery. The haematoxylin-eosin (HE) staining of the tumour showed differently sized lumina lined by flat or cubic epithelial cells (Fig. 1C). These lumina were filled with PAS-positive colloid. Initial histological differential diagnoses considered in our case were ectopic lacrimal gland [3] or sweat gland and iris adenoma [4, 5], which could be excluded upon further histological und immunohistochemical examination. Indeed, immunohistochemical staining showed that the lining cells were positively stained with antibodies against cytokeratin AE1/AE3 (cytokeratin 1–8, 10, 14–16 and 19), cytokeratin 18 and BerEP4. To confirm our final diagnosis of an ectopic thyroid tissue, we conducted immunohistochemistry using anti-thyroid-transcripting-factor-1 (TTF-1) and anti-thyroglobulin antibodies staining the tumour cells positive (Fig. 1D). Another differential diagnosis was a metastasis of a previously unknown follicular thyroid carcinoma. However, both the histologically benign aspect and the medical history (tumour known at least since childhood, multiple strumectomies without histological evidence of malignancy) did not support the latter differential diagnosis. Nevertheless, further examination of the thyroid was recommended to exclude a malignant tumour with certainty.

After uncomplicated surgery, the patient last presented to the clinic in mid-2020. The patient reported a subjective improvement in vision, but visual acuity was still poor compared to the left eye.

In the clinical examination, visual acuity had already increased from perception of hand motion preoperatively to 0.25 (equivalent to 20/80) with autorefraction. The still reduced visual acuity was attributed to uncorrected irregular corneal astigmatism after penetrating keratoplasty and advanced optic nerve damage from known open angle glaucoma. The corneal graft was clear and the two continuous sutures were tight. The anterior segment was free of irritation, the temporal iridectomy was open and the intraocular lens was in place. There was no evidence of tumour recurrence. The intraocular pressure was normal under treatment with travoprost on both eyes.

In addition, tumour screening was performed in the meantime to exclude metastasis of a previously unknown follicular thyroid carcinoma. The sonography and scintigraphy of the thyroid showed bilateral struma recurrence, which was more pronounced on the right side, without evidence of autonomy or nodules. Under levothyroxine substitution, the patient showed a euthyroid metabolic state (thyroid-stimulating hormone in the low normal range, as desired). Overall, the findings were stable compared to the previous findings in 2013.

## Discussion and conclusions

Ectopic thyroid tissue in the iris, also known as a thyroid glandular epithelial choristoma of the iris, has only been described twice in the literature. The first case was described in 2006 in a fifteen-year-old boy [1]. During the ophthalmological examination, a multinodular pink tumour had been found in the iridocorneal angle. No further pathological changes were observed. The tumour was asymptomatic and surgically removed to exclude malignancy. Histologically, positive staining with antibodies against TTF-1 and thyroglobulin was found here, as well.

The second case was published in 2019 describing a 76-year-old male patient with an iris mass in the anterior chamber since birth [2]. It had recently increased in size and surgical removal of the mass was performed. Immunohistochemistry once again confirmed the diagnosis of a thyroid glandular epithelial choristoma of the iris.

In contrast to the cases described so far, the ectopic tissue in our case led secondarily to endothelial compensation and visual deterioration. Consequently, the tissue was resected en bloc during the subsequent keratoplasty. Endothelial decompensation and visual deterioration might have been caused by cataract surgery and iStent® implantation, complicated by the extensive tumour in the anterior chamber with anterior synechiae. As a limitation, increased intraocular pressure may also have contributed to corneal decompensation and visual deterioration, and these cannot be attributed directly and exclusively to ectopic thyroid tissue. Nevertheless, this case provides new insights into a clinical picture that has been rarely described to date.

In conclusion, we report the third case of ectopic thyroid tissue in the iris. Both previous cases remained asymptomatic, whereas in our case, size and location of the ectopic thyroid tissue might have contributed to a more complex cataract surgery resulting in endothelial decompensation. Therefore, in such cases appropriate patient information should be provided prior to cataract surgery. Furthermore, when ectopic thyroid tissue is detected in the anterior chamber of the eye, careful histological examination and tumour screening is important to exclude malignant diagnoses such as a metastasis of a follicular thyroid carcinoma.

## Data Availability

Data sharing is not applicable to this article as no datasets were generated or analysed during the current study.

## Abbreviations

HE: Haematoxylin-Eosin
TTF-1: Anti-thyroid-transcripting-factor-1
